# Ferroportin deficiency drives olaparib resistance in ovarian cancer via the cGAS–STING–STAT3 survival axis

**DOI:** 10.1002/ctm2.70885

**Published:** 2026-11-02

**Authors:** Jiagui Song, Zhengyang Guo, Decao Yang, Ping Yu, Yinjia Li, Zhuhui Yuan, Xiaojuan Ma, Xianlong Li, Hongyan Guo, Lixiang Xue, Tong Liu

**Affiliations:** ^1^ Center of Basic Medical Research Institute of Medical Innovation and Research Peking University Third Hospital Beijing China; ^2^ Cancer Center of Peking University Third Hospital Beijing China; ^3^ Beijing Key Laboratory of Interdisciplinary Research in Gastrointestinal Oncology (BLGO) Peking University Third Hospital Beijing China; ^4^ Department of Obstetrics and Gynecology Peking University Third Hospital Beijing China; ^5^ Department of Radiation Oncology, Cancer Center of Peking University Third Hospital Peking University Third Hospital Beijing China

**Keywords:** cGAS–STING, ferroptosis, FPN, Olaparib resistance

## Abstract

**Background:**

Ovarian cancer (OC) remains the most lethal gynaecological malignancy, and acquired resistance to PARP inhibitors such as olaparib represents a major clinical challenge. Although dysregulation of iron metabolism has been implicated in tumorigenesis, the specific role of the iron exporter *SLC40A1* (ferroportin, FPN) in mediating olaparib resistance remains poorly understood. The study aimed to elucidate the clinical significance of FPN deficiency and to explore therapeutic strategies for reversing olaparib resistance.

**Methods:**

FPN expression was evaluated in OC samples from a clinical cohort of 19 patients, stratified by olaparib responsiveness, to determine its prognostic value. Functional mechanisms were investigated using multiple OC cell lines, patient‐derived organoids (PDOs), and mouse xenograft models. We integrated analyses of iron homeostasis, ROS dynamics, DNA repair capacity, and innate immune signaling, with a focus on the cGAS–STING–STAT3 signaling cascade. The therapeutic potential of combining olaparib with the ferroptosis inducer erastin or the STING inhibitor C‐176 was assessed as a strategy to overcome FPN deficiency‐driven resistance.

**Results:**

Clinically, FPN expression was significantly downregulated in olaparib‐resistant tissues compared with sensitive controls (*p* < 0.05), and low FPN levels were strongly associated with poor therapeutic outcomes. Mechanistically, FPN deficiency promoted intracellular iron accumulation and ROS generation, which led to increased ferroptosis vulnerability, impaired homologous recombination repair (HRR) and concomitant activation of the cGAS–STING–STAT3 inflammatory axis, thereby driving olaparib resistance. Notably, in vivo studies demonstrated that combining olaparib with either erastin or C‐176 significantly suppressed tumour growth and restored drug sensitivity compared with monotherapy (*p* < 0.01), supporting the translational potential of targeting this axis.

**Conclusions:**

Our study establishes a clinically relevant mechanistic connection between ferroportin deficiency and cGAS–STING–STAT3‐mediated olaparib resistance in OC. We identify the FPN–ROS–cGAS–STING–STAT3 axis as an actionable vulnerability in resistant OC, indicating that this pathway is a promising therapeutic target to overcome olaparib resistance.

**Highlights:**

- FPN loss drives olaparib resistance and predicts poor outcomes in ovarian cancer.
- FPN loss causes iron and ROS accumulation, impairing HRR and activating the cGAS–STING–STAT3 axis.
- The cGAS–STING–STAT3 pro‐survival program drives olaparib resistance.
- STING inhibition restores olaparib sensitivity in resistant ovarian cancer.

## Graphical abstract

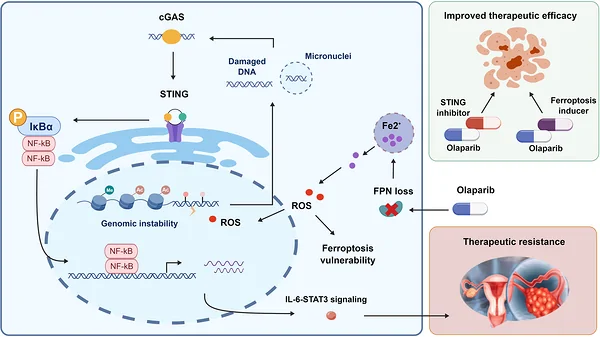

## 1 INTRODUCTION

Among gynaecologic malignancies, ovarian cancer (OC) continues to account for the highest mortality, primarily due to late‐stage diagnosis and poor prognosis following metastatic spread.^1^, ^2^ Despite upfront surgical debulking followed by carboplatin/paclitaxel, roughly 70% of patients ultimately develop relapsing disease.^3^, ^4^ These clinical realities underscore an unmet need for strategies that can overcome or prevent treatment failure.

Olaparib, a poly (ADP‐ribose) polymerase (PARP) inhibitor, exerts its cytotoxic effect by trapping PARP enzymes on DNA and converting single‐strand breaks into double‐strand breaks during replication ‐ a process particularly lethal in cells with defective homologous recombination repair (HRD).^5^, ^6^, ^7^, ^8^ PARP inhibitors have become standard maintenance therapy for advanced ovarian cancer, yet their clinical impact is curtailed by inherent or acquired resistance: ∼40% of patients ultimately relapse, and response rates in platinum‐refractory settings remain modest (5%–30%).^9^ Elucidating resistance mechanisms and devising strategies to resensitize tumours therefore represent pressing clinical priorities.^5^, ^10^

Genomic instability frequently gives rise to micronuclei that rupture, releasing nuclear DNA into the cytosol where it activates the cGAS–STING pathway ‐ an innate immune sensing cascade with paradoxical roles in oncology.^11^, ^12^ It can promote tumour suppression by triggering autophagy, apoptosis, and NF‐κB/SASP‐mediated immune responses.^13^, ^14^ Conversely, chronic STING stimulation provokes ER stress, sustained type I interferon signaling, and non‐canonical NF‐κB activation.^15^ Such prolonged signaling fosters an immunosuppressive microenvironment that facilitates immune evasion and therapeutic resistance. Moreover, activation of the non‐canonical NF‐κB pathway promotes tumour metastasis and the release of IL‐6, which directly supports tumour cell survival through the IL‐6/STAT3 signaling axis. This mechanism can occur under various stress conditions, such as radiation and chemotherapy drugs.^16^ PARP inhibitors (such as olaparib) exert their antitumour effects through inhibiting replication functions, but concurrently activate the cGAS‐STING signaling pathway, which contributes to tumour cell apoptosis and shapes antitumour immune responses through inflammation signaling.^17^, ^18^ However, whether cGAS–STING pathway activation contributes to olaparib resistance remains to be fully elucidated.

Cellular iron handling profoundly influences OC progression and treatment outcomes. Ferroportin (FPN), encoded by *SLC40A1*, is the only identified iron efflux channel and is indispensable for iron homeostasis.^19^, ^20^, ^21^ Downregulation of FPN is a recurrent feature of OC relative to adjacent normal tissue, resulting in aberrant iron retention.^22^ Conversely, elevated SLC40A1 has been associated with improved OC patient survival.^23^ The resulting iron overload fuels proliferative signaling (e.g., Wnt/beta‐catenin) while simultaneously sensitizing cells to ferroptosis ‐ an iron‐dependent form of regulated necrosis driven by lipid peroxidation.^24^ Whether FPN loss specifically contributes to PARP inhibitor resistance, however, has not been adequately addressed.

In this study, we established that FPN deficiency in OC leads to iron overload and consequent genomic instability, which chronically activates the cGAS–STING axis. Rather than inducing tumour cell death, this activated cGAS–STING signaling functions as a compensatory survival mechanism, allowing cancer cells to tolerate the replication stress induced by olaparib and thereby developing drug resistance. Using 3D cell cultures, patient‐derived organoids (PDOs), and in vivo mouse models, we demonstrate that low FPN expression correlates with olaparib resistance. We provide evidence that the combination of ferroptosis inducers or STING inhibitors with olaparib effectively disrupts this survival axis, offering a promising precision medicine strategy to overcome PARPi resistance in OC.

## 2 RESULTS

### 2.1 SLC40A1 (FPN) expression is correlated with olaparib resistance in ovarian cancer

To explore the mechanistic basis of olaparib resistance, we first assessed the inherent drug responsiveness across a panel of widely used cell lines. We determined olaparib IC50 values in A2780, OVCAR8 and SKOV3 cells (Figure 1A–F) and treated these cell lines with graded concentrations of olaparib (Figure S1A–C). SKOV3 exhibited the highest IC50 and was therefore classified as the most resistant line. This ranking was corroborated by exposing the three cell lines to 100 µM olaparib for 48 h: SKOV3 retained a fully viable morphology, whereas A2780 and OVCAR8 displayed a marked loss of viability (Figure 1A, C, E; Figure S1A–C).

**FIGURE 1:**
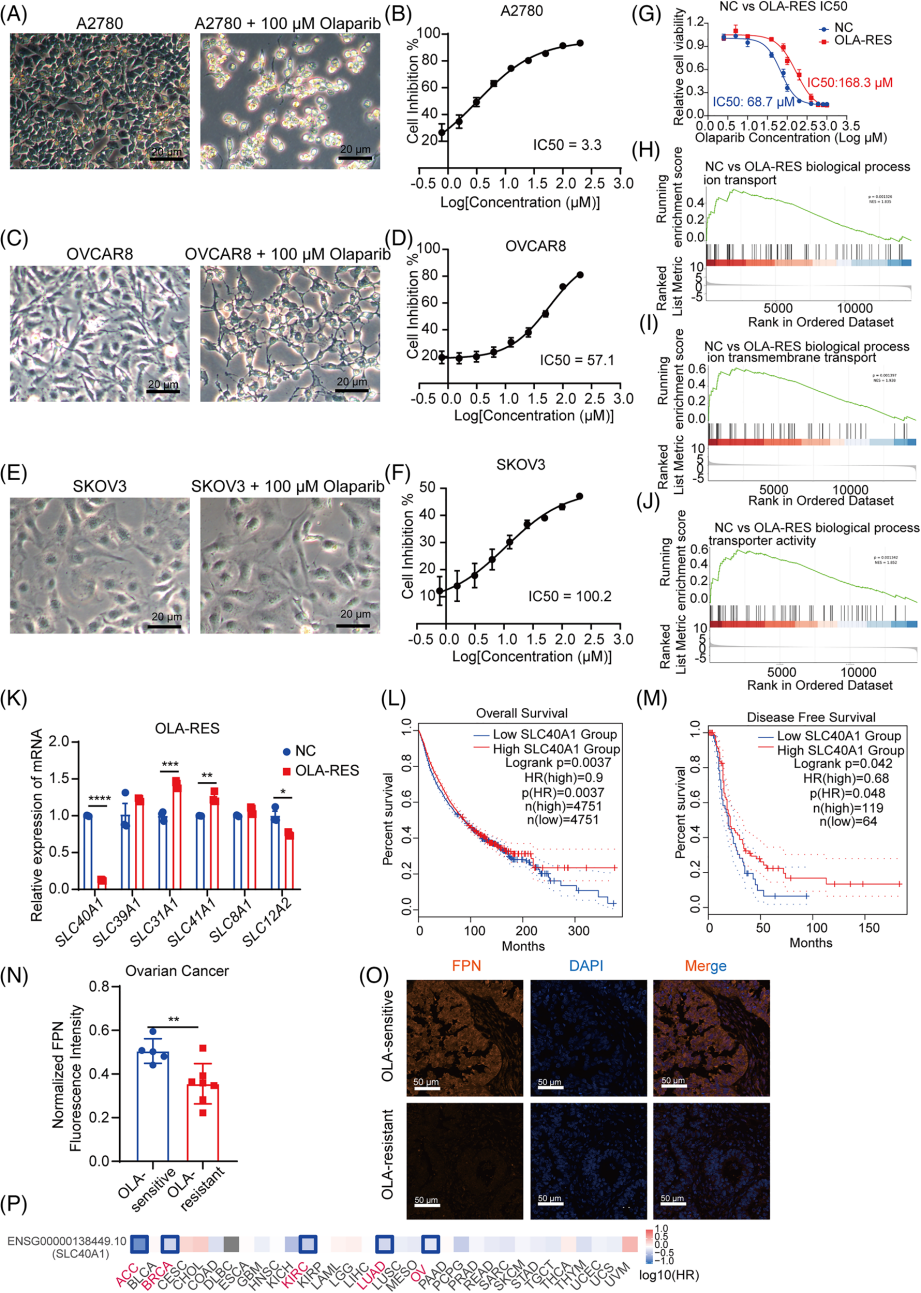
*SLC40A1* (FPN) expression is correlated with olaparib resistance in ovarian cancer. (A–F) CCK‐8 assays were performed to determine cell viability in A2780, SKOV3 and OVCAR8 cell lines treated with graded concentrations of olaparib for 48 h (B, D and F). IC_50_ values were calculated using GraphPad Prism (*n* = 3). Representative images of A2780, SKOV3 and OVCAR8 cells after olaparib (100 µM) treatment for 48 h (A, C and E). (G) Establishment of drug‐resistant cell lines by olaparib dose escalation; the IC_50_ values for the parental and OLA‐RES cells were calculated using GraphPad Prism. (H, I and J) GSEA of the biological processes ‘ion transport’, ‘ion transmembrane transport’ and ‘transporter activity’ based on RNA‐seq data in NC and OLA‐RES cells. (K) Total RNA was extracted from NC and OLA‐RES cells treated with 100 µM olaparib, and the mRNA levels of *SLC40A1*, *SLC39A1*, *SLC31A1*, *SLC12A1* and *SLC8A2* were quantified by qPCR. (L) Kaplan–Meier overall survival (OS) analysis of ovarian cancer patients stratified by *SLC40A1* expression levels. Patients were classified into high‐expression (top 30%) and low‐expression (bottom 30%) groups using the GEPIA 2 database. (M) Kaplan–Meier disease‐free survival analysis of all OC samples classified on the basis of the *SLC40A1* expression level (cut‐off high = 70%, cut‐off low = 30%), performed via the GEPIA 2 database. (*p < 0.05; **p < 0.01; ***p < 0.001; ns, not significant). The data are presented as the mean ± s.e.m. One‐way ANOVA with Tukey's multiple comparisons test (A). (N) Clinical ovarian cancer samples were collected from the Department of Obstetrics and Gynaecology at our institution, comprising 19 cases: 5 olaparib‐sensitive, 7 olaparib‐resistant, 4 platinum‐sensitive, and 3 platinum‐resistant. Opal staining and quantitative analysis were performed to evaluate the association between FPN expression and olaparib resistance. (O) Representative Opal staining images of FPN in olaparib‐sensitive and olaparib‐resistant ovarian cancer tissues. (P) According to the GEPIA 2 database, *SLC40A1* exhibits different expression across 33 tumour types.

To uncover the molecular basis of this differential sensitivity, we generated olaparib‐resistant OVCAR8 cells (named OLA‐RES) and performed unbiased pathway analysis (Figure 1G). RNA‐seq analysis comparing NC and OLA‐RES upon olaparib treatment revealed significant enrichment of signatures related to ion transport, ion transmembrane transporter activity, and transporter activity (Figure 1H–J). We further validated the expression changes of several ion transporters by qPCR, among which *SLC40A1* showed the most pronounced alteration and was significantly downregulated in resistant cells (Figure 1K). FPN, the protein encoded by *SLC40A1*, represents the only known iron exporter. These findings provide evidence that FPN loss may contribute to olaparib resistance.

Analysis using the GEPIA 2 database, comprising 9502 cancer patients with overall survival (OS) data and 183 OC patients with disease‐free survival (DFS) data, revealed that lower *SLC40A1* expression was significantly associated with poorer survival outcomes (Figure 1L, M; Figure S1D). Moreover, Opal immunohistochemical staining of 19 OC samples including olaparib‐sensitive (*n* = 5), olaparib‐resistant (*n* = 7), cisplatin‐sensitive (*n* = 4) and cisplatin‐resistant (*n* = 3) specimens demonstrated that low FPN expression was significantly correlated with olaparib resistance (Figure 1N, O, Table S1), but not cisplatin resistance (Figure S1D). This prognostic trend extended to adrenocortical carcinoma (ACC), breast invasive carcinoma (BRCA), kidney renal clear‐cell carcinoma (KIRC), lung adenocarcinoma (LUAD) and ovarian cancer (OV) (Figure 1P). Collectively, these findings establish a strong association between FPN expression and olaparib resistance.

### 2.2 FPN expression dictates olaparib sensitivity

Having established that FPN deficiency correlates with olaparib resistance in OC, we next asked whether basal FPN abundance predicts intrinsic drug sensitivity across distinct OC models. CCLE data showed that basal *SLC40A1* abundance is lowest in SKOV3 among the three lines tested (Figure 2A), a result we validated at both mRNA and protein levels (Figure 2B, C). Three‐dimensional spheroid cultures recapitulated the hierarchy observed in 2D: SKOV3 formed the densest spheres and simultaneously displayed the weakest FPN signal (Figure 2D, E).

**FIGURE 2:**
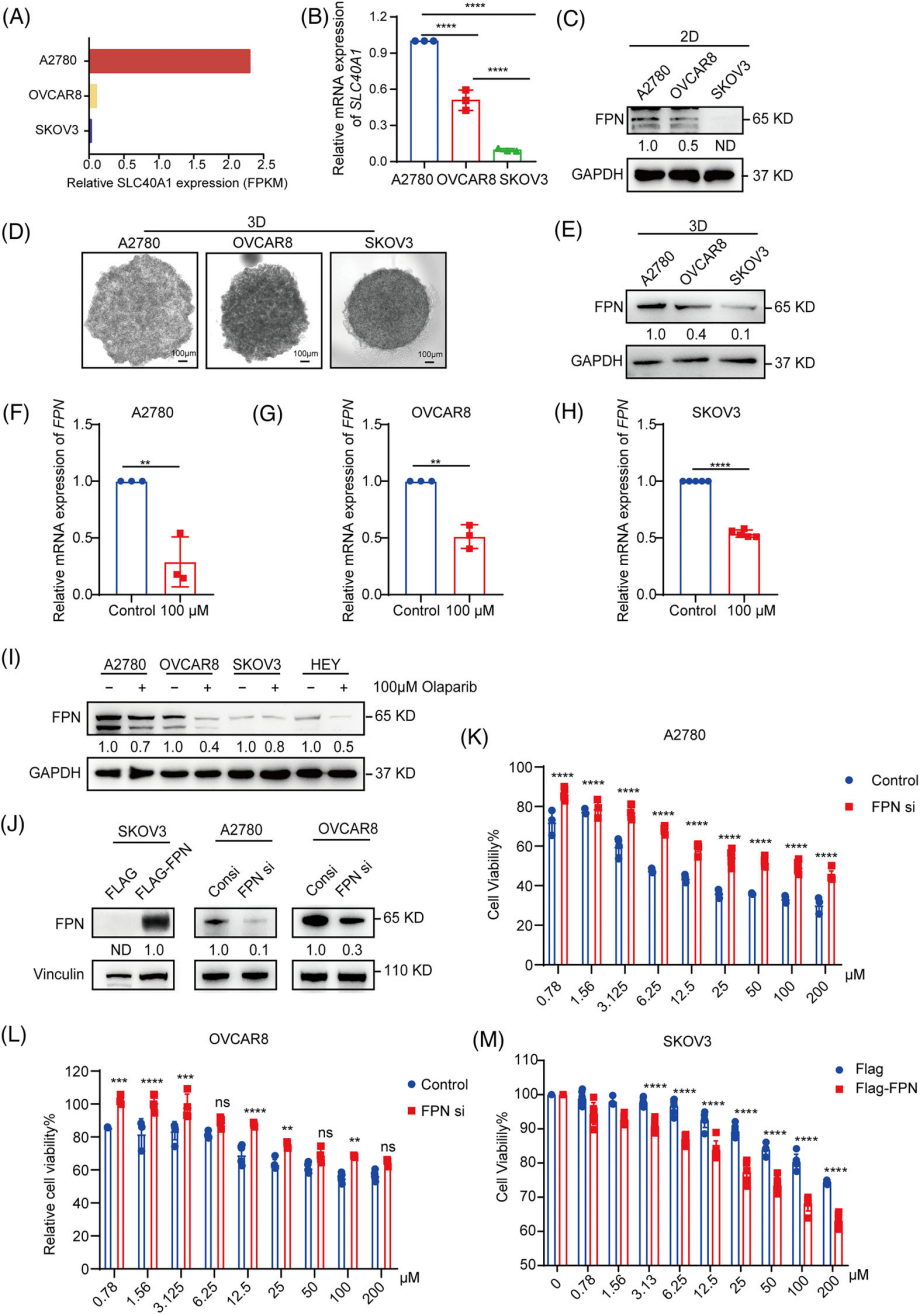
FPN expression dictates olaparib sensitivity. (A) The CCLE database shows the expression levels of *SLC40A1* in A2780, SKOV3 and OVCAR8. (B) mRNA levels of *SLC40A1* in A2780, SKOV3 and OVCAR8 were quantified by qPCR. (C) Western blotting validated FPN expression levels in A2780, SKOV3 and OVCAR8. (D) The hanging drop method was used to assess the 3D spheroid‐forming ability of A2780, SKOV3 and OVCAR8. Representative images of 3D spheroids formed by A2780, OVCAR8 and SKOV3 cells are shown. (E) The expression levels of FPN in A2780, SKOV3 and OVCAR8 3D spheroids were verified by western blotting. (F, G and H) qPCR was used to detect the mRNA expression level of *SLC40A1* in A2780, SKOV3, and OVCAR8 cells treated with 100 µM olaparib. (I) The protein level of FPN in A2780, SKOV3 and OVCAR8 cells treated with 100 µM olaparib was verified by western blotting. (J) The knockdown efficiency of FPN in A2780 and OVCAR8 cells, and the FPN overexpression efficiency in SKOV3 cells, were verified by western blotting. (K and L) CCK‐8 assays were performed to assess the sensitivity of A2780 and OVCAR8 cells to olaparib after FPN knockdown. (M) A CCK‐8 assay was performed to assess the sensitivity of SKOV3 cells to olaparib upon FPN overexpression.

To further dissect this relationship, we examined whether olaparib treatment itself alters FPN expression. Notably, exposure to olaparib suppressed FPN expression in four OC cell lines (Figure 2F–I), suggesting that the drug induces an FPN‐related mechanism that enhances iron retention and, consequently, resistance.

In A2780 and OVCAR8 lines, which intrinsically express high levels of ferroportin, FPN depletion significantly elevated olaparib tolerance across a wide concentration range, indicating enhanced resistance (Figure 2J–L). Conversely, overexpression of FPN in the inherently resistant, FPN‐low SKOV3 line sensitized the cells to olaparib, as evidenced by a marked reduction in cell viability under olaparib treatment (Figure 2J, M). These gain‐ and loss‐of‐function experiments show that FPN serves as a key positive regulator of olaparib sensitivity in OC.

### 2.3 FPN deficiency primes ovarian cancer cells for ferroptosis

As the sole known mammalian iron exporter, FPN constrains the labile iron pool (LIP); its loss elevates intracellular Fe^2^^+^, drives lipid peroxidation and precipitates ferroptosis. To test whether this biology modulates PARP‐inhibitor efficacy, we generated OVCAR8 cell lines stably expressing shRNA against *SLC40A1* (shFPN) or a non‐targeting control (shNC) (Figure 3A) and performed rescue experiments. Olaparib‐induced cytotoxicity was rescued by ferroptosis inhibitors (Ferr‐1) and deferoxamine (DFO), but not by inhibitors of apoptosis or necrosis (Figure S2A). We examined whether FPN deficiency alters cellular sensitivity to ferroptosis. As shown, erastin—a canonical ferroptosis inducer—showed a stronger inhibitory effect on the viability of shFPN cells compared with shNC controls (Figure 3B, C). Concordantly, FPN knockdown increased the transcriptional signature of ferroptosis, *PTGS2* mRNA level (Figure 3D), as well as lipid peroxidation (Figure 3E, F). These results demonstrate that FPN deficiency renders OC cells hypersensitive to ferroptosis induction.

**FIGURE 3:**
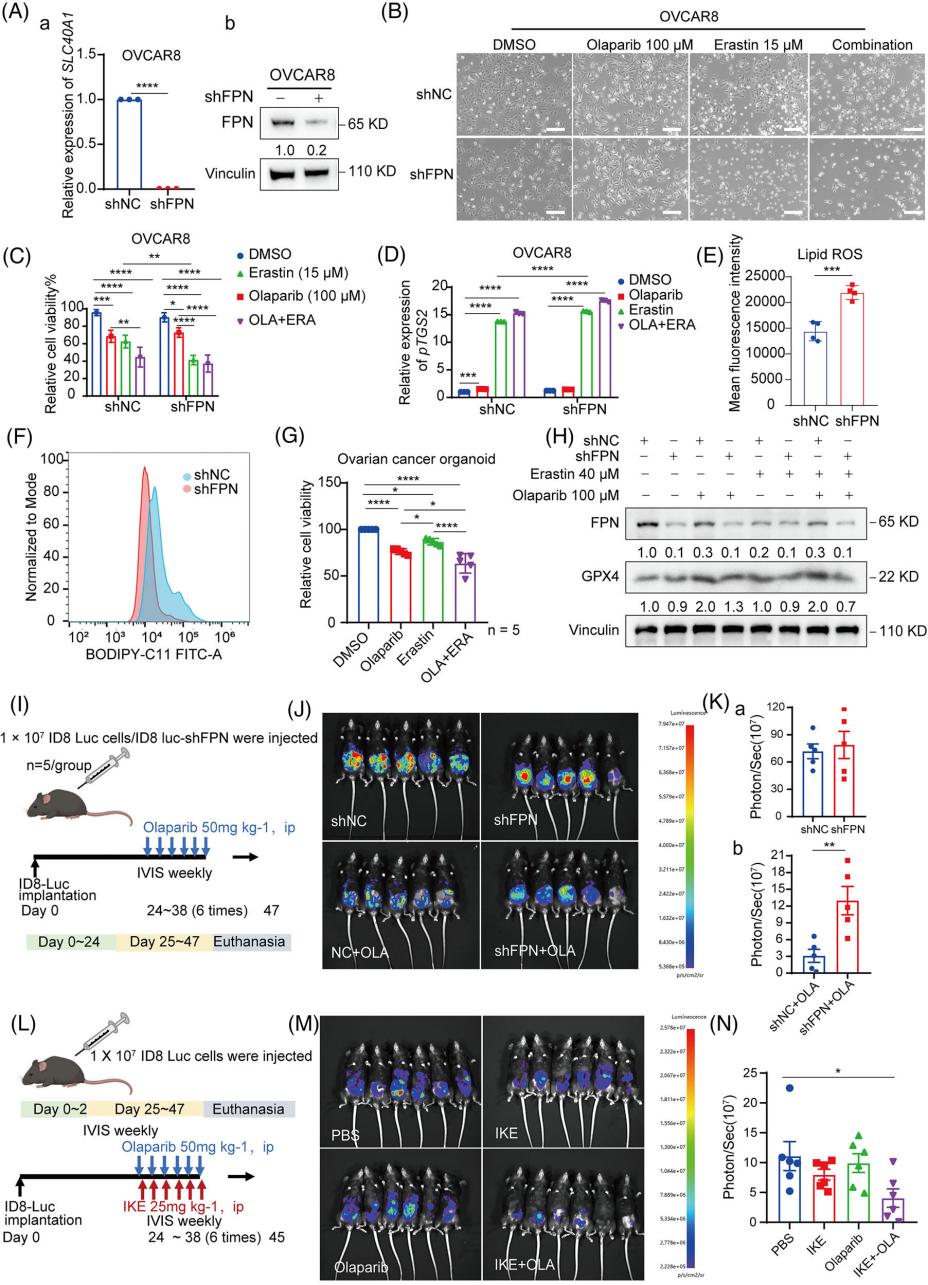
FPN deficiency primes ovarian cancer cells for ferroptosis. (A) mRNA level of *SLC40A1* in shNC and shFPN cells, as quantified by qPCR (*n* = 3) (a); the knockdown efficiency of FPN in OVCAR8 cells was verified by western blotting (b). (B) Representative phase‐contrast images of OVCAR8 parental cells and FPN knockdown cells treated with erastin (15 µM) and olaparib (100 µM) as indicated for 48 h. (C) Corresponding quantification of cell viability is shown, as determined by CCK‐8 assay. (D) mRNA level of *PTGS2* in shNC and shFPN cells, as quantified by qPCR (*n* = 3). (E and F) Flow cytometric analysis of LPO levels in shNC and shFPN cells. Fluorescence signals were normalized to unstained control, and the cell counts were normalized. Statistical significance was determined relative to the control group. (G) Relative viability of ovarian cancer organoids treated with erastin (ferroptosis inducer) and olaparib, as measured by the CellTiter‐Glo 3D Cell Viability Assay (*n* = 5). (H) shNC and shFPN cells were treated with erastin (15 µM) and olaparib (100 µM) as indicated for 48 h. The expression levels of FPN, GPX4 and vinculin were verified by western blotting. (I) Schematic diagram of the tumour‐bearing experiment with olaparib (50 mg/kg) treatment in ID8‐Luc shNC / ID8‐Luc shFPN cell xenografted mice. (J and K) Bioluminescence images (J) and distribution of photon counts (K: a and b) of shNC (*n* = 5) and shFPN (*n* = 5) xenografted mice. (L) Schematic diagram of the tumour‐bearing experiment with IKE (25 mg/kg) and olaparib (50 mg/kg) in ID8‐Luc cell‐xenografted mice. (M and N) Bioluminescence images (M) and distribution of photon counts (N) of ID8‐Luc (*n* = 5) xenografted mice.

Building on this ferroptosis‐primed state, we next evaluated whether concurrent ferroptosis induction could enhance therapeutic efficacy. Pairing erastin with olaparib produced a profound inhibitory effect on OC cells and organoids (Figure 3B, C, G) and reduced GPX4 protein levels in the combined treatment group (Figure 3H). The same phenomenon was recapitulated in murine ID8 cells (Figure S2B–F) and human CAOV3 cells (Figure S2G–I). In accord with our data that FPN knockdown led to a marked increase in intracellular Fe^2^^+^ levels compared with the control (Figure S2J,K), these results suggest that FPN deficiency may promote ROS accumulation by blocking iron efflux, thereby rendering OC cells more susceptible to ferroptosis induction.

We further extended these findings to mouse models. Female C57BL/6 mice received intraperitoneal ID8‐Luc cells carrying either shNC or shFPN. Bioluminescence imaging (IVIS) showed that FPN knockdown promotes tumour survival under olaparib treatment (Figure 3I–K). To test whether pharmacological ferroptosis activation resensitizes tumours, we substituted erastin with IKE (a metabolically stable erastin analogue). Following randomization into vehicle, olaparib, IKE, or combination groups, mice implanted with ID8‐Luc cells were monitored. The combined treatment strategy achieved superior tumour regression compared to any single‐agent group (Figure 3L–N).

Collectively, these data establish FPN as a molecular rheostat linking iron homeostasis to ferroptosis sensitivity, and provide a preclinical rationale for combining ferroptosis inducers with olaparib in FPN‐low OCs. However, a paradox emerged: although the erastin–olaparib combination effectively suppressed tumour growth in vivo, the combination index (CI) in vitro did not indicate synergistic cytotoxicity (Figure S2L). More importantly, given that FPN deficiency primes cells for ferroptosis, it remains unclear why FPN‐low cells exhibit resistance to olaparib monotherapy rather than enhanced sensitivity. This contradiction suggests that FPN loss may simultaneously activate a compensatory survival pathway that counteracts ferroptotic vulnerability and drives drug resistance. Therefore, we next sought to investigate alternative mechanisms underlying FPN‐mediated olaparib resistance.

### 2.4 FPN deficiency suppresses homologous recombination repair and is associated with olaparib resistance

We previously established an olaparib‐resistant cell line (OLA‐RES) that exhibits significant resistance to olaparib (Figure 1G). Notably, OLA‐RES cells also exhibit reduced FPN expression (Figure 4A). RNA‐seq data revealed that the homologous recombination repair (HRR) pathway was suppressed in OLA‐RES cells (Figure 4B). To determine whether FPN deficiency contributes to this phenomenon, we performed RNA‐seq analysis comparing shNC and shFPN cells. FPN knockdown significantly downregulated the expression of key HRR‐related genes, including *BRCA1, BRCA2, RAD50*, and *ATM* (Figure 4C).

**FIGURE 4:**
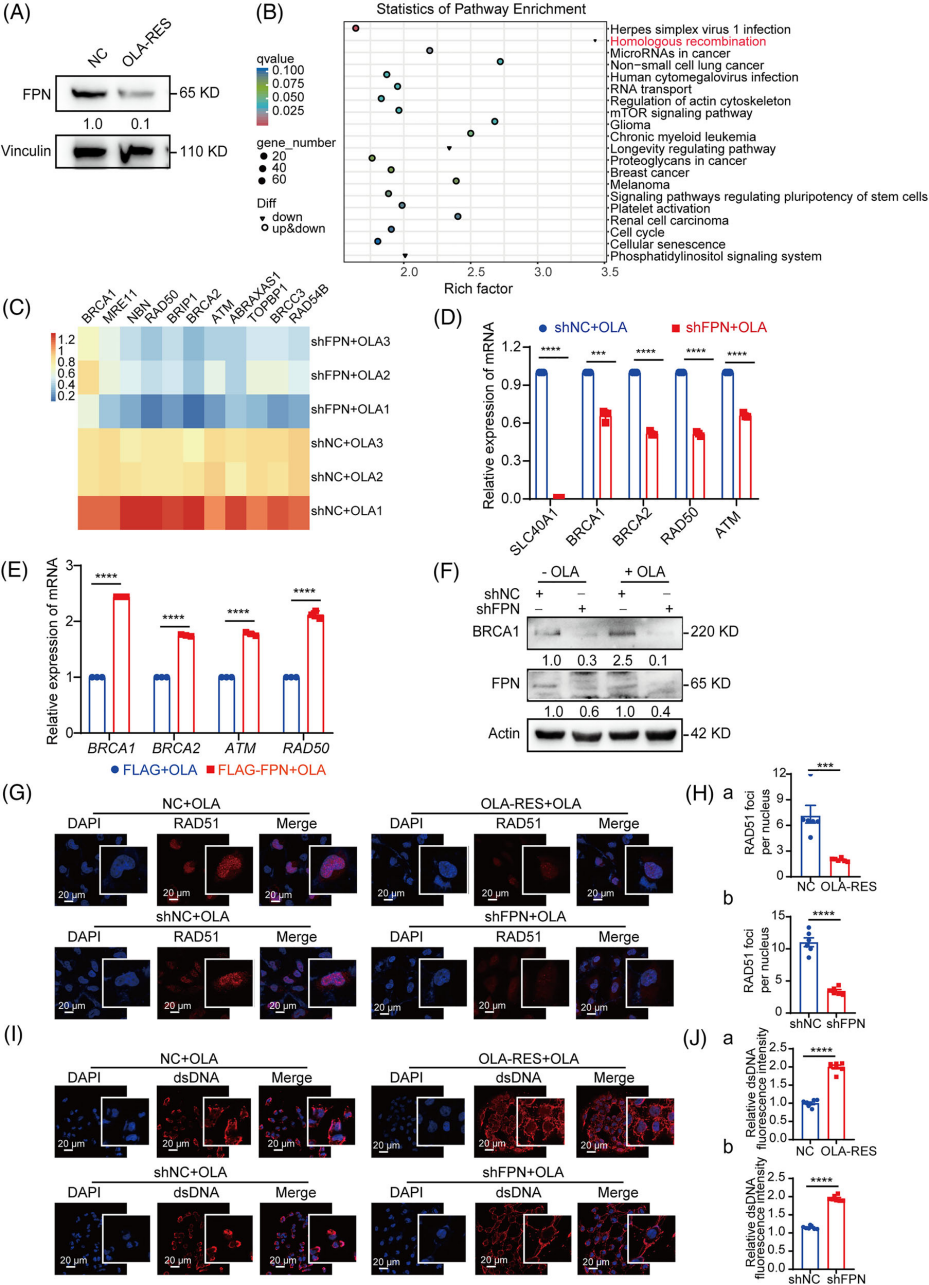
FPN deficiency suppresses HRR and is associated with olaparib resistance. (A) The protein level of FPN in NC and OLA‐RES cells was determined by western blotting. (B) KEGG pathway enrichment of significantly differentially expressed genes in OLA‐RES cells. (C) Heatmap (log2 fold change) of homologous recombination‐associated genes in shNC and shFPN cells treated with 100 µM olaparib for 48 h. (D) Expression of homologous recombination‐associated genes in shNC and shFPN cells treated with 100 µM olaparib for 48 h, as quantified by qPCR. (E) Expression of homologous recombination‐associated genes in Flag‐NC and Flag‐FPN cells treated with 100 µM olaparib for 48 h, as quantified by qPCR. (F) The protein level of BRCA1 in shNC and shFPN cells was determined by western blotting. (G) RAD51 foci assay. Immunofluorescence staining was performed on NC, OLA‐RES and shNC/shFPN cells following treatment with 100 µM olaparib. RAD51 was stained with an anti‐RAD51 antibody and an Alexa Fluor 594 immunofluorescence secondary antibody. (H: a, b) RAD51 foci were quantified using ImageJ software for fluorescence intensity and foci density, and the number of foci per cell was counted (*n* = 6). (I) Immunofluorescence staining was performed on NC, OLA‐RES and shNC/shFPN cells following treatment with 100 µM olaparib. dsDNA was stained with an anti‐dsDNA antibody and an Alexa Fluor 594 immunofluorescence secondary antibody. (J: a, b) Mean fluorescence intensity was quantified using ImageJ software (*n* = 7).

We validated these findings by qPCR in both shFPN and Flag‐FPN overexpression cells, demonstrating that HRR gene expression was positively correlated with FPN levels (Figure 4D, E). Consistently, western blotting confirmed that FPN knockdown suppressed BRCA1 protein expression (Figure 4F). Collectively, these results indicate that FPN deficiency impairs HRR at both the mRNA and protein levels. Paradoxically, despite the transcriptional suppression of HRR genes, OLA‐RES cells exhibit enhanced olaparib resistance, a phenomenon that suggests olaparib‐resistant cells may bypass HRR‐dependent DNA repair through alternative survival pathways, thereby driving drug resistance.

In line with this, the RAD51 foci assay revealed that OLA‐RES cells formed fewer RAD51 foci than controls following olaparib treatment; similarly, FPN knockdown recapitulated this phenotype (Figure 4G, H). These observations suggest that olaparib effectively inhibits HRR function. Meanwhile, we observed that OLA‐RES cells accumulated markedly more intracellular double‐strand DNA (dsDNA) than controls following olaparib treatment; again, FPN knockdown produced a similar outcome (Figure 4I, J). These data indicate that FPN deficiency induced nuclear envelope rupture and DNA leakage into the cytoplasm, correlated with olaparib resistance.

### 2.5 FPN deficiency activates the cGAS–STING–IL‐6–STAT3 pathway to drive olaparib resistance

Intracellular accumulation of dsDNA may initiate cGAS–STING‐dependent signaling. This hypothesis was supported by RNA‐seq data from FPN‐deficient and OLA‐RES cells showing the upregulation of key genes within the cGAS–STING axis and subsequent IL6‐STAT3 pathway, specifically *TMEM173* (STING)*, MB21D1* (cGAS)*, TBK1, IL6, and STAT3* (Figure 5A, B). These findings imply that FPN deficiency activates the cGAS‐STING‐IL6‐STAT3 pathway, which in turn could maintain the viability of OLA‐RES cells through an alternative mechanism. IKBKE elevation reflects STING‐driven NF‐κB signaling, given its established role as a downstream effector kinase of STING. Subsequently, we performed validation experiments. qPCR analysis revealed that compared with the control cells, treating OLA‐RES cells with olaparib significantly upregulated the expression of *TMEM173* (STING) by 4.65‐fold (Figure 5C). Similarly, FPN knockdown led to increased *TMEM173* (STING) expression, whereas FPN overexpression had the opposite effect (Figure 5D, E). Western blotting further confirmed that OLA‐RES cells exhibited not only decreased FPN expression but also enhanced STING expression (Figure 5F).  Consistently, FPN overexpression in OLA‐RES cells also downregulated STING expression and attenuated STING signaling (Figure S3A). Conversely, in cells with FPN overexpression, phosphorylated IκBα levels were diminished, and STING signaling was correspondingly suppressed (Figure 5F).

**FIGURE 5:**
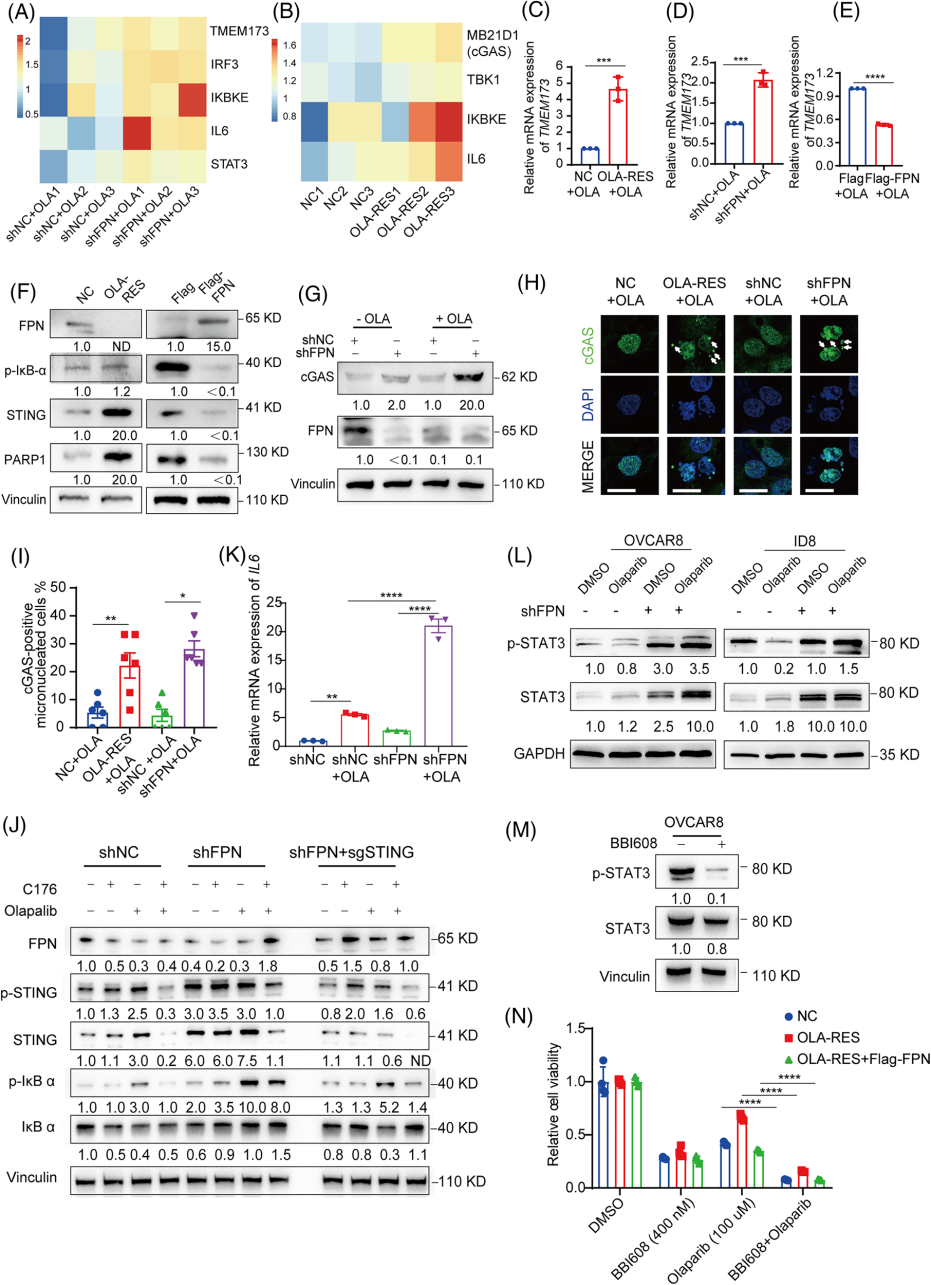
FPN deficiency activates the cGAS–STING/IL6–STAT3 pathway to drive olaparib resistance. (A) Heatmap (log2 fold change) of cGAS–STING‐associated genes in shNC and shFPN cells treated with 100 µM olaparib for 48 h. (B) Heatmap (log2 fold change) of cGAS–STING‐associated genes in NC and OLA‐RES cells treated with 100 µM olaparib for 48 h. (C) mRNA level of *TMEM173* in NC and OLA‐RES cells treated with 100 µM olaparib for 48 h, as detected by qPCR (*n* = 3). (D) mRNA level of *TMEM173* in Flag‐NC and Flag‐FPN cells treated with 100 µM olaparib for 48 h, as quantified by qPCR (*n* = 3). (E) mRNA level of *TMEM173* in shNC and shFPN cells treated with 100 µM olaparib for 48 h, as quantified by qPCR (*n* = 3). (F) Homologous recombination‐associated proteins and STING expression in NC and OLA‐RES cells treated with 100 µM olaparib for 48 h, as measured by western blotting. (G) Homologous recombination‐associated proteins and STING expression in Flag‐NC and Flag‐FPN cells treated with 100 µM olaparib for 48 h, as detected by western blotting. (H and I) Cells were stained with an anti‐cGAS antibody followed by an Alexa Fluor 488 immunofluorescence secondary antibody. (J) shNC, shFPN and shFPN/STING KO cells were treated with the STING inhibitor C‐176 (1.5 µM), olaparib (100 µM), or the combination; phospho‐STING/STING and phospho‐IκBα/IκBα were detected by western blotting as indicated. (K) mRNA level of IL‐6 in shNC and shFPN cells treated with 100 µM olaparib for 48 h, as quantified by qPCR (*n* = 3). (L) STAT3, phospho‐STAT3 Tyr705 in shNC and shFPN cells of OVCAR8 (L, left) and ID8 (L, right) treated with 100 µM olaparib for 48 h, as detected by western blotting. (M) NC and OLA‐RES cells were treated with the STAT3 inhibitor BBI‐608 at 400 nM for 48 h; western blotting was performed as indicated. (N) NC, OLA‐RES and OLA‐RES/Flag‐FPN cells were treated with BBI‐608 (400 nM), olaparib (100 µM), or the combination; cell viability was evaluated by a CCK‐8 assay.

Upon comparing the olaparib‐treated shNC and shFPN groups, we observed that cGAS was elevated in the shFPN group, particularly following olaparib treatment when FPN expression was further reduced (Figure 5G). Collectively, these findings demonstrate that FPN deficiency activates the cGAS‐STING pathway by blocking iron efflux and thereby promoting ROS accumulation, a well‐established driver of genomic instability (Figure 3 and Figure S2). Strikingly, we found that cGAS colocalized with micronuclei surrounding the nucleus, with the highest number of cGAS‐positive micronuclei observed in shFPN cells (Figure 5H, I). This indicates that FPN deficiency induces nuclear DNA leakage, thereby triggering the cGAS‐STING pathway. Building on the findings reported by Hong et al. (2022),^16^ which demonstrated that the cGAS–STING pathway activated IL6‐STAT3 signaling through the NF‐κB pathway to promote tumour cell survival, we further evaluated STING activation using C‐176, a STING inhibitor. As shown in Figure 5J and Figure S3B, elevated ratios of phospho‐STING/STING and phospho‐IκBα/IκBα confirmed robust STING pathway activation induced by FPN deficiency and olaparib treatment. In contrast, transfection with STING sgRNA (sgSTING) suppressed STING expression and activation of the downstream pathway. Given that sustained STING activation can drive tumour cell survival, proliferation, and therapeutic resistance via non‐canonical mechanisms, including the NF‐κB/IL‐6/STAT3 cascade, we proceeded to examine IL6 levels. Our results revealed a more than 20‐fold elevation in IL‐6 expression under conditions of low FPN expression (Figure 5K). Moreover, phosphorylated STAT3 levels were markedly elevated, an observation consistent across both OVCAR8 and ID8 cells (Figure 5L). Treatment with the STAT3 inhibitor BBI608 eliminates cellular resistance to olaparib (Figure 5 M, N).

Together, these data reveal that FPN deficiency, in the context of olaparib resistance and following the failure of HRR, leads to genomic instability and nuclear DNA leakage. This, in turn, activates the cGAS‐STING cascade and its subsequent IL6–STAT3 axis, ultimately promoting the survival of drug‐resistant cells.

### 2.6 Targeting STING synergizes with olaparib to overcome resistance

To further substantiate that FPN deficiency induces olaparib resistance through the STING pathway, we employed the STING inhibitor C‐176. Our results demonstrated that co‐treatment with olaparib and a low concentration of C‐176 (1.5 µM) significantly reduced the IC50 of OVCAR8 cells from 87.47 to 37.53 µM (Figure 6A). This effect was even more pronounced in shFPN cells, in which the combination of olaparib with low‐concentration C‐176 decreased the IC50 from 109.9 to 22.99 µM (Figure 6B). The CI of the combination of olaparib and C‐176 confirmed synergistic effects in both shNC and shFPN groups (Figure 6C), indicating that inhibiting the STING pathway markedly augments the therapeutic potency of olaparib. We corroborated this finding not only in cellular models but also in OC organoids derived from three patients, in which the combination of olaparib and C‐176 produced greater cytotoxicity than either agent alone (Figure 6D).

**FIGURE 6:**
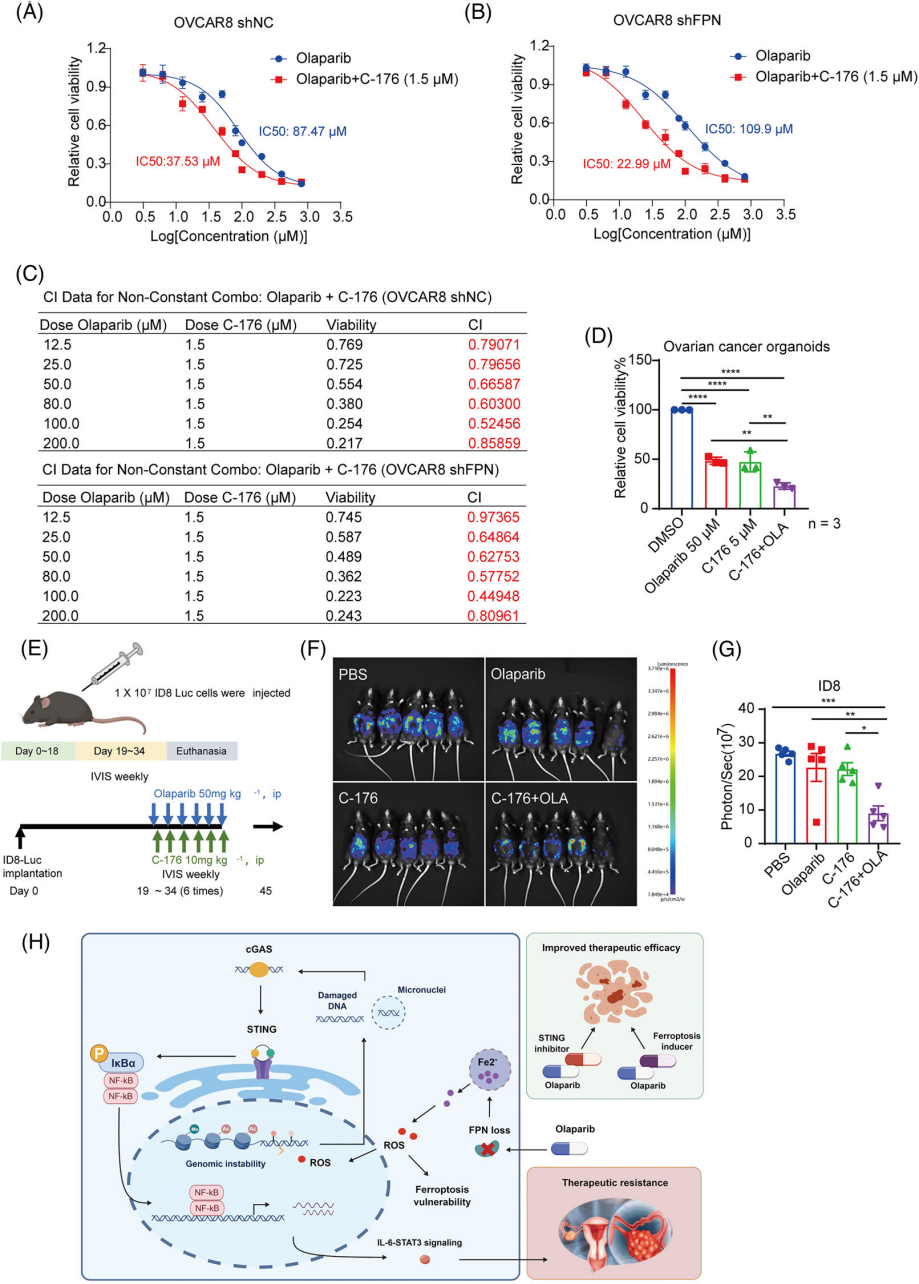
Targeting STING synergizes with olaparib to overcome resistance. (A and B) Relative cell viability of OVCAR8 shNC and shFPN cells treated with C‐176 (1.5 µM) and olaparib for 48 h, as measured by CCK‐8 assays (*n* = 5); the IC_50_ values were calculated using GraphPad Prism. (C) The combination index (CI) of olaparib and C‐176 was calculated using CompuSyn software. (D) Relative viability of ovarian cancer organoids treated with C‐176 (STING inhibitor) and olaparib, as measured by the CellTiter‐Glo 3D Cell Viability Assay (*n* = 3). (E) Schematic diagram of the tumour‐bearing experiment with olaparib (50 mg/kg) and C‐176 (10 mg/kg) treatment in ID8‐Luc shNC cell xenografted mice. (F and G) Bioluminescence images (F) and distribution of photon counts (G) of shNC (*n* = 5) xenografted mice. (H) Working model. Low expression of FPN leads to iron buildup within ovarian cancer cells. Although this iron accumulation does not abrogate viability, it heightens ROS levels and renders cells more vulnerable to ferroptosis. Consequently, pairing a ferroptosis inducer with olaparib should produce synergistic tumour killing. Moreover, FPN deficiency drives PARPi resistance via the cGAS–STING/IL‐6–STAT3 axis, indicating that pharmacological inhibition of this pathway could restore olaparib sensitivity.

To provide more definitive evidence for this conclusion, we extended these findings to an in vivo model. Female C57BL/6 mice received an abdominal injection of ID8‐Luc cells and olaparib, C‐176, or the combination as indicated. Bioluminescence imaging (IVIS) revealed that, compared with either monotherapy, the combination of olaparib and C‐176 exhibited significantly enhanced anti‐tumour activity (Figure 6E‐G).

Collectively, these findings, corroborated by cellular models, patient‐derived organoids, and in vivo studies, underscore that inhibiting the STING pathway can overcome olaparib resistance and markedly enhance its therapeutic efficacy (Figure 6H).

## 3 Discussion

### 3.1 Principal findings

Despite the clinical success of PARP inhibitors (PARPis) such as olaparib as maintenance therapy, the inevitable emergence of acquired resistance remains the principal obstacle to long‐term disease control. Approximately 40% of patients eventually relapse after developing PARPi resistance, and no standard‐of‐care strategy currently exists to reverse this phenotype. Combinatorial strategies involving agents such as durvalumab and cediranib have been employed to circumvent olaparib resistance, while the precise molecular mechanisms driving this resistance remain undefined.^5^, ^25^ Here, we identify ferroportin (FPN) deficiency as a key driver of olaparib resistance and delineate a cGAS‐STING–STAT3 survival axis that can be therapeutically targeted. Unlike previous studies that primarily focused on the role of iron metabolism in proliferation or ferroptosis susceptibility, our research highlights the critical role of a non‐canonical ‘FPN–cGAS–STING–IL6’ signaling axis. FPN loss creates a ‘double‐edged sword’: it induces a vulnerability to ferroptosis but simultaneously triggers a cGAS–STING–IL6‐dependent survival program that overrides DNA damage toxicity. These findings suggest that targeting this compensatory STING‐IL6 pathway can re‐sensitize resistant cells, potentially offering a new combinatorial strategy to extend the clinical benefit of PARP inhibitors.

### 3.2 Critical Analysis

Our observation aligns with the work of Basuli et al. (2017), confirming that FPN loss leads to intracellular iron overload and renders cells susceptible to ferroptosis. We observed that combining olaparib with ferroptosis inducers (e.g., erastin) did not show strong synergistic effects. This creates a paradox: if FPN deficiency promotes ferroptosis, why do patients still relapse? Critically, this divergence may be explained by the activation of a powerful, adaptive survival mechanism that counteracts the pro‐death signals. While iron overload generates large amounts of reactive oxygen species (ROS) and DNA damage, our findings reveal that this genomic instability serves as a trigger for the cGAS‐STING axis.^22^, ^26^

Furthermore, while the cGAS‐STING cascade is classically known for initiating anti‐tumour immunity, our results indicate that in the context of olaparib resistance, it mediates a tumour‐intrinsic survival signal. This aligns with recent findings by Hong et al. (2022) regarding chromosomal instability.^16^ This distinction is crucial because it implies that the cGAS‐STING signaling axis functions as a context‐dependent ‘molecular switch’. In our model, FPN deficiency‐induced chromosomal instability leads to cytosolic DNA leakage, which activates cGAS‐STING to drive the transcription of IL‐6 via NF‐κB. The subsequent activation of the IL‐6/STAT3 axis provides a robust bypass mechanism, allowing tumour cells to withstand olaparib treatment despite HRR defects. This provides a more nuanced understanding of why simply inducing DNA damage is insufficient and why targeting the downstream IL‐6/STAT3 cascade is necessary to overcome resistance.

### 3.3 Potential Mechanisms

The biological plausibility of our findings lies in the direct link between iron metabolism, genomic instability, and innate immune sensing. It is possible that olaparib treatment itself downregulates FPN expression, creating a feedback loop that exacerbates intracellular iron overload. Crucially, to address how this high intracellular iron and ROS environment leads to the transcriptional repression of HR‐related genes like BRCA1, BRCA2 and ATM, we hypothesize the involvement of specific molecular bridges. First, excessive ROS may directly oxidize and inhibit key transcription factors, such as E2F1, which are essential for maintaining the basal expression of BRCA1 and BRCA2.^27^ Second, as iron serves as an essential cofactor for various epigenetic modifiers (e.g., TET demethylases and JmjC‐domain histone demethylases), iron overload could dysregulate these enzymes, leading to aberrant DNA methylation or repressive histone modifications at the promoters of HR genes.^28^ Third, chronic oxidative stress may activate stress‐responsive kinases (e.g., p38 MAPK) that actively repress the transcription of DNA repair factors as part of a metabolic adaptation. This iron‐addicted state promotes oxidative stress and impairs HRR, leading to the emergence of micronuclei accompanied by the release of nuclear DNA into the cytosol. This aligns with the ‘non‐canonical function’ theory of cGAS‐STING, suggesting that in the context of chronic CIN, the pathway defaults to promoting inflammation‐mediated survival. Cytosolic DNA is recognized by cGAS, activating STING and TBK1, which consequently activates the NF‐κB signaling pathway to drive IL‐6 transcription. The upregulation of IL‐6 further activates the STAT3 pathway, a known promoter of cell survival and drug resistance. Our use of the STING inhibitor C‐176, which successfully re‐sensitized resistant cells in both organoid and in vivo models, supports the hypothesis that this axis is a druggable target.^16^, ^29^ However, the clinical translation of STING inhibition requires careful navigation of the pathway's dual nature. It is well‐established that while chronic, low‐level STING activation drives tumour progression (as observed in our FPN‐deficient models), acute and robust STING signaling is essential for initiating anti‐tumour immunity via Type I interferon production. Consequently, systemic administration of STING inhibitors carries the theoretical risk of dampening systemic immune surveillance. To reconcile this dichotomy, future clinical strategies should place emphasis on developing tumour‐targeted delivery systems (e.g., nanoparticle‐encapsulated STING inhibitors) to ensure high local concentrations within the tumour microenvironment while minimizing exposure to systemic immune cells. Alternatively, combining STING inhibition with immune checkpoint blockade (ICB) could help preserve the host's anti‐tumour immune competence while blocking the pro‐tumourigenic survival signals described in our study.

### 3.4 Research Strengths

Our study includes the comprehensive elucidation of the FPN–cGAS–STING–IL6 axis, moving beyond simple correlation to establish mechanistic causality through multidimensional validation. We utilized a robust multi‐model approach, including RNA‐seq analysis, patient‐derived organoids, and in vivo xenografts, which offers higher clinical relevance compared with traditional 2D cell lines. Furthermore, the identification of a synergistic strategy combining olaparib with a STING inhibitor (C‐176) provides immediate translational value for overcoming PARPi resistance.

### 3.5 Our Limitations

Our study has several limitations that warrant mention to ensure a comprehensive interpretation of the results:

First, the preclinical nature of our study limits our ability to infer causality in a human clinical setting. While our organoid and mouse models provide robust evidence, and we analysed some clinical samples, we lacked a large‐scale clinical cohort to validate the correlation between FPN levels, cGAS–STING activation and patient prognosis.

Second, we focused primarily on tumour‐intrinsic mechanisms. Further investigation is needed to elucidate the systemic immune effects of the cGAS‐STING pathway on the tumour immune microenvironment (TIME), which is a known confounder in STING‐related research.

Finally, while we identified the IL‐6/STAT3 axis as a downstream effector, the precise upstream epigenetic regulation of SLC40A1 (FPN) by olaparib (e.g., the hypermethylation mentioned in our results) requires deeper exploration to fully understand the initiation of this resistance loop.

Subsequent investigations should focus on prospective studies using immunocompetent models to dissect the interplay between this pathway and the immune microenvironment, as well as early‐phase clinical trials to validate the efficacy and safety of combining PARPi with STING or IL‐6 inhibitors in patients with FPN‐deficient OC.

## 4 CONCLUSION

Overall, this study addresses the challenging scientific issue of resistance to olaparib, a PARP inhibitor that serves as the last line of defence in the clinical treatment of OC. Through multidimensional validation, we have established that FPN deficiency is a key driver of olaparib resistance. The loss of FPN not only sensitizes cells to ferroptosis but also restricts HRR and initiates the cGAS‐STING pathway. This, in turn, promotes tumour cell survival via the IL‐6/STAT3 signaling axis, thereby reinforcing resistance (Figure 6H). These findings offer a new theoretical basis for the precision treatment of OC and propose novel strategies to overcome resistance by targeting iron metabolism and the cGAS–STING pathway, ultimately offering the potential to improve the prognosis of patients.

## 5 MATERIALS AND METHODS

Specific methods used in this study are described below, for other routine experimental procedures, please refer to the Supporting Materials and Methods.

### 5.1 Chemical compounds

Pharmacological agents were procured as follows: erastin (S7242), IKE (S8877), Ferrostatin‐1/Fer‐1 (S7243), DFO (S6849), Z‐VAD‐FMK (S7023), and Necrostatin‐1/Nec‐1 (S8037) from Selleck Technologies; olaparib (S1060) and C‐176 (S6575) for in vitro experiments from Selleck; and olaparib (T3015), BBI608 (T3218) and C‐176 (T5154) for in vivo studies from Targetmol.

### 5.2 Construction of olaparib resistance (OLA‐RES) cells

To establish drug‐resistant cell lines, OVCAR8 cells were gradually adapted to olaparib (S1060) through stepwise concentration increases (2–50 µM), with 2–3 passages per step to establish stable resistant lines. The cells that survived at each concentration were considered to be drug‐resistant cells at that concentration. Ultimately, the cells that survived treatment with 50 µM olaparib were designated as OLA‐RES.

### 5.3 Cell samples preparation for RNA‐seq analysis

The OVCAR8 NC and OLA‐RES cells, as well as shNC and shFPN cells, were plated in 10 cm dishes to ∼80% confluence. Subsequently, the cells were exposed to either DMSO or 100 µM olaparib for 48 h. Following treatment, they were rinsed twice with PBS, and 1 mL of TRIzol was added per plate for cell collection. The collected samples were then transported on dry ice to a commercial provider for RNA‐seq analysis. The raw data (sequencing) generated for this study have been deposited in the BIG Submission (BIG Sub) with accession number PRJCA074919 at the website of https://ngdc.cncb.ac.cn/gsub/submit/bioproject/list.

### 5.4 3D Hanging Drop Culture for Tumour Spheroid Formation

Spheroids were generated using the hanging‐drop technique: logarithmic‐phase OC cells (A2780, OVCAR8, SKOV3) were resuspended to 5 × 10^3^ cells/mL with complete medium. Then 20 µL aliquots were dispensed onto the inner surface of inverted 10‐cm dish lids. Lids were sealed over dishes containing 10 mL PBS (humidified chamber) and incubated at 37°C, 5% CO_2_ for 72 h. Resulting spheroids were collected by pipetting drops into tubes or multi‐well plates.

### 5.5 Organoid preparation and culture

Patient‐derived OC tissues were collected and minced into fine fragments under sterile conditions, followed by enzymatic digestion using a solution supplemented with Collagenase IV and DNase I at 37°C for 30–60 min. The digested cell clusters were filtered through a 70 µm strainer, centrifuged, and resuspended in basement membrane extract (BME). The cell‐BME suspension was plated as droplets in a pre‐warmed culture plate and polymerized at 37°C. Each well was then overlaid with a defined human organoid culture medium, consisting of Advanced DMEM/F‐12 supplemented with EGF, Noggin, and R‐spondin‐1 and small‐molecule inhibitors (such as A83‐01 and Y‐27632). The culture medium was refreshed every 48–72 h to support organoid proliferation.

### 5.6 Organoid chemosensitivity assay

Dissociated OC organoids at passage 1 were collected and resuspended in cold Basement Membrane Extract (BME). The suspension was seeded into a pre‐warmed 96‐well plate at 2 × 10^3^ cells per well and maintained to polymerize at 37°C for 15 min. Following polymerization, each well was overlaid with 100 µL of complete organoid culture medium. Following 24 h incubation, cells were refreshed with new medium containing the chemotherapeutic agents (e.g., olaparib, erastin, C176) or vehicle control (e.g., DMSO). Following a 3‐day‐treatment period, organoid viability was quantified by CellTiter‐Glo® 3D, Promega. The luminescence signal for each well was read on a microplate reader, and cell viability data were processed with GraphPad Prism.

### 5.7 Real‐time quantitative PCR (real‐time qPCR)

Real‐time qPCR was performed using SYBR qPCR Master Mix (Vazyme) and the Applied Biosystems QuantStudio 5 system (Applied Biosystems). mRNA level detection, total RNA was extracted from the cells with TRIzol (Invitrogen) and reverse transcribed into cDNA (Vazyme). Beta‐actin (ACTB) was used as a control to normalize the expression levels. The primers for qPCR are listed in Table 1.

**TABLE 1:** The primers utilized for qPCR.

Target	Primer sequence
*IL6* F primer	5‘CTGAAGGAGCAGAGGCAACT 3’
*IL6* R primer	5‘GGTGGAGCTGACAATGAGGT3’
*TMEM173* F primer	5‘ACTGTGGGGTGCCTGATAAC3’
*TMEM173* R primer	5‘TGGCAAACAAAGTCTGCAAG3’
*PTGS2* F primer	5‘CTTCACGCATCAGTTTTTCAAG3’
*PTGS2* R primer	5‘TCACCGTAAATATGATTTAAGTCCAC3’
*SLC40A1* F primer	5‘CTAGAGGATCTATTTCCGGTGAATTCA3’
*SLC40A1* R primer	5‘AGTCGGATCCAACAACAGATGTATTTG3’
*Actin* F primer	5‘CCGGACACGGTCACCCGTTG3’
*Actin* R primer	5‘CGCCTTGCACGCTAGCTGGT3’
*BRCA1* F primer	5‘GGTGGTACATGCACAGTTGC3’
*BRCA1* R primer	5‘ACTCTGGGGCTCTGTCTTCA3’
*BRCA2* F primer	5‘AGCTCTTCACCCTGCAAAAA3’
*BRCA2* R primer	5‘CCAATGCCTCGTAACAACCT3’
*ATM* F primer	5‘GGACAGTGGAGGCACAAAAT3’
*ATM* R primer	5‘GTGTCGAAGACAGCTGGTGA3’
*RAD50* F primer	5‘CTTGGATATGCGAGGACGAT3’
*RAD50* R primer	5‘CCAGAAGCTGGAAGTTACGC3’
*SLC39A1* F primer	5‘CCTCACACTCCCATGGTGGAG3’
*SLC39A1* R primer	5‘GCTGTATTGATCACCAAACCGG3’
*SLC31A1* F primer	5‘CCAGTCACCGTGGAGAACAG3’
*SLC31A1* R primer	5‘GCTCCTCTTCCACTCAGCAG3’
*SLC41A1* F primer	5‘CCTGGAACTGAATCACTGGCGATAC3’
*SLC41A1* R primer	5‘ACCTCCCTTGTGGCTGGACTTC3’
*SLC8A1* F primer	5‘GTGTTTGTCGCTCTTGGAACCTC3’
*SLC8A1* R primer	5‘CGTTGCTTCCGGTGACATTG3’
*SLC12A2* F primer	5‘AAGGGCAGCGAGGAAGCCAA3’
*SLC12A2* R primer	5‘GCCGTTCTGGAAGCTCACGTT3’

### 5.8 Lipid peroxidation assay

The indicated cells were plated at 3×10^5^ per well in 6‐well plates 24 h prior to treatment (triplicate wells). Upon attachment, cells received the indicated treatments. Following treatment, cells were stained with fresh medium containing 0.1× BODIPY 581/591 C11 dye (Invitrogen, D3861). After a 30‐min incubation at 37°C in a humidified atmosphere with 5% CO_2_, cells were washed with PBS and detached by trypsinization to generate a single‐cell suspension. We quantified lipid peroxidation via flow cytometry on a Beckman Coulter CytoFLEX, with data processed using FlowJo.

### 5.9 FerroOrange staining for labile iron detection

Cells were plated in fluorescence culture dishes and incubated overnight at 37°C with 5% CO_2_. After removing the culture medium, cells underwent three washes with Hanks' Balanced Salt Solution (HBSS) or phenol red‐free serum‐free medium. Following drug treatment, the medium was removed and cells were washed three times with HBSS. FerroOrange working solution (1 µM) was freshly prepared by diluting the 1 mM stock solution in HBSS or phenol red‐free serum‐free medium immediately before use. Cells were exposed to 1 µM FerroOrange working solution at 37°C for 30 min in the dark. After staining, cells were directly observed under a fluorescence microscope without washing (excitation/emission ≈ 543/580 nm).

### 5.10 Tumour xenograft and therapeutic intervention in mice

Specific pathogen‐free (SPF) female C57BL/6 mice (6–8 weeks, 20–22 g) were sourced from Vital River Laboratory Animal Technology. Mice were inoculated intraperitoneally with 1 × 10^7^ ID8 cells to establish a disseminated peritoneal tumour model that recapitulates key clinical features of human OC. Drug treatment was initiated 24 days after tumour cell inoculation, and the cell dose, administration route, and timing were based on our previously published protocol (PMID: 40818202). Mice were randomly assigned to treatment groups, including control, olaparib (50 mg/kg), IKE (25 mg/kg), C‐176 (10 mg/kg), olaparib plus IKE, or olaparib plus C‐176. All agents were administered intraperitoneally every 2 days for 2 weeks. Tumour burden was monitored every two days by an observer blinded to treatment assignment, using digital calliper measurements and changes in abdominal perimeter. Cage positions were rotated regularly to minimize environmental bias. Mice were euthanized after 10 days of tumour progression assessment, and no animals or data points were excluded from the analysis.

## AUTHOR CONTRIBUTIONS

T.L., J.S. and Z.G. conceived and designed the study. T.L., J.S., Z.G., Y.L., X.M., Z.Y. and D.Y. performed the experiments and analyzed the data. P.Y. collected clinical samples and patient data. L.X., H.G., and X.L. contributed to data interpretation and manuscript revision. T.L. supervised the study and finalized the manuscript. All authors read and approved the final version.

## CONFLICT OF INTEREST STATEMENT

The authors declare no conflicts of interest.

## ETHICS STATEMENT

Animal experiments were approved by the Animal Welfare and Ethics Committee of Peking University Third Hospital, with approval numbers LA2023031. OC tissues are clinical surgery samples with informed consent from Peking University Third Hospital, and was approved by Medical Ethics committee of Peking University Third Hospital with approval number LM2024117 (for organoids).

## Supporting information



Supporting Information: ctm270885‐sup‐0001‐TableS1.docx

Supplementary Figure 1. (A, B, C) A2780 (A), OVCAR8 (B), and SKOV3 (C) cells treated with olaparib at the indicated concentrations, as analysed by the IncuCyte S3 system (*n* = 5). (D) The expression of *SLC40A1* in 88 cases of normal ovarian tissues and 426 cases of ovarian cancer tissues, as analysed via the GEPIA 2 database. (E) Opal staining and quantitative analysis were performed to evaluate the association between FPN expression and platinum resistance from 4 platinum‐sensitive, and 3 platinum‐resistant OC samples.

Supplementary Figure 2. (A) OVCAR8 cell lines stably expressing shRNA against SLC40A1 (shFPN) or a non‐targeting control (shNC) were treated with olaparib (100 µM) as indicated for 48 h. Cells were co‐treated with the ferroptosis inhibitor DFO (10 µM), apoptosis inhibitor Z‐VAD‐FMK (5 µM) and necrosis inhibitor Nec‐1 (20 µM). (B) Representative phase‐contrast images of ID8 parental cells and FPN knockdown cells treated with erastin (15 µM) and olaparib (100 µM) as indicated for 48 h. (C) (a) mRNA level of FPN in shNC and shFPN cells, as quantified by qPCR (*n* = 3); (b) the knockdown efficiency of FPN in ID8 cells was verified by western blotting. (D) ID8 cell viability was detected by a CCK‐8 assay following treatment with erastin (15 µM), olaparib (100 µM), or the combination. (E) mRNA level of *PTGS2* in shNC and shFPN cells, as detected by qPCR (*n* = 3). (F) shNC and shFPN ID8 cells were treated with erastin (15 µM) and olaparib (100 µM) as indicated for 48 h. The expression levels of FPN, GPX4, and Vinculin were verified by western blotting. (G) The knockdown efficiency of FPN in CAOV3 cells was verified by western blotting. (H) CAOV3 cell viability was measured by a CCK‐8 assay following treatment with erastin (10 µM), olaparib (100 µM), or the combination. (I) shNC and shFPN CAOV3 cells were treated with erastin (10 µM) and olaparib (100 µM) as indicated for 48 h. The expression levels of FPN, GPX4, and Vinculin were verified by western blotting. (J‐K) Representative confocal images of labile iron levels detected by FerroOrange staining. Scale bars: 100 µm (overview) and 20 µm (zoom‐in). Each experiment was performed with 4 to 5 replicate wells and was repeated 2 or 3×. Data are presented as mean ± SEM. Statistical significance was determined by one‐way ANOVA with Sidak's multiple comparisons test. (L) The combination index (CI) of olaparib and erastin, corresponding to Figure 3C, was calculated using CompuSyn software.

Supplementary Figure 3. (A) FPN, STING, and indicated proteins in NC, OLA‐RES, OLA‐RES‐sgSTING, and OLA‐RES‐FlagFPN cells were detected by western blotting as indicated. (B) shNC, shFPN, and shFPN/STING KO cells were treated with the STING inhibitor C‐176 (1.5 µM), olaparib (100 µM), or the combination; phospho‐TBK1/TBK1 and phospho‐IRF3/IRF3 were detected by western blotting as indicated.

Supporting Information: ctm270885‐sup‐0005‐SuppMat.docx

## Data Availability

The RNA‐seq data generated in this study have been deposited in BIG Submission with accession number PRJCA074919. All the data are available upon request to the corresponding authors. GEPIA 2 was accessed at https://gepia2.cancer‐pku.cn/#index and The Cancer Cell Line Encyclopedia (CCLE) database is available at https://sites.broadinstitute.org/ccle.
