# Supplementary material for: Ferroportin deficiency drives olaparib resistance in ovarian cancer via the cGAS–STING–STAT3 survival axis
Source: Clin Transl Med. 2026 Nov 2;16(11):e70885. doi: 10.1002/ctm2.70885 (PMC13725362; doi:10.1002/ctm2.70885)
Supplement: Supplementary file 1 [file CTM2-16-e70885-s003.docx]

**Supplementary Table 1. Clinical information of patients with ovarian cancer.**

| **Number** | **Staging** | **Age** | **BMI** | **Tumor burden** | **Chemosensitivity** | **Pausimenia（Yes=1，No=0）** | **Hypertension（Yes=1，No=0）** |
| --- | --- | --- | --- | --- | --- | --- | --- |
| **1** | **III** | **40** | **25.3** | **medi** | **Cisplatin-sensitive** | **0** | **0** |
| **2** | **III** | **65** | **28.12** | **high** | **Cisplatin-sensitive** | **1** | **0** |
| **3** | **IV** | **69** | **30.07** | **high** | **Cisplatin-resistant** | **1** | **1** |
| **4** | **III** | **69** | **21.63** | **high** | **Cisplatin-resistant** | **1** | **0** |
| **5** | **IV** | **61** | **28.04** | **high** | **Cisplatin-resistant** | **1** | **0** |
| **6** | **IC1** | **75** | **22.99** | **high** | **Olaparib-sensitive** | **1** | **1** |
| **7** | **III** | **52** | **27.18** | **low** | **Olaparib-sensitive** | **0** | **0** |
| **8** | **III** | **65** | **22.55** | **low** | **Cisplatin-sensitive** | **1** | **0** |
| **9** | **III** | **54** | **27.2** | **medi** | **Cisplatin-sensitive** | **1** | **0** |
| **10** | **III** | **53** | **27.73** | **high** | **Olaparib-resistant** | **1** | **0** |
| **11** | **III** | **40** | **21.9** | **medi** | **Olaparib-resistant** | **0** | **0** |
| **12** | **III** | **55** | **21.2** | **high** | **Olaparib-resistant** | **1** | **0** |
| **13** | **III** | **36** | **25.53** | **high** | **Olaparib-resistant** | **0** | **0** |
| **14** | **III** | **55** | **27.05** | **high** | **Olaparib-resistant** | **0** | **0** |
| **15** | **III** | **34** | **27.3** | **high** | **Olaparib-resistant** | **0** | **0** |
| **16** | **II** | **65** | **20.7** | **high** | **Olaparib-resistant** | **1** | **0** |
| **17** | **IC2** | **65** | **21.7** | **low** | **Olaparib-sensitive** | **1** | **0** |
| **18** | **IA** | **57** | **17.9** | **low** | **Olaparib-sensitive** | **1** | **0** |
| **19** | **IC3** | **53** | **28.04** | **low** | **Olaparib-sensitive** | **1** | **0** |
