# Supplementary material for: Ferroportin deficiency drives olaparib resistance in ovarian cancer via the cGAS–STING–STAT3 survival axis
Source: Clin Transl Med. 2026 Nov 2;16(11):e70885. doi: 10.1002/ctm2.70885 (PMC13725362; doi:10.1002/ctm2.70885)
Supplement: Supplementary file 2 [file CTM2-16-e70885-s004.docx]

**Supplementary Materials and Methods**

**Cell Culture**

Validated cell lines, including OVCAR8 (CVCL_1629), A2780 (CVCL_0134), SKOV3 (CVCL_0532), ID8 (CVCL_IU14), and CAOV3 (CVCL_0201), were sourced from the Cell Resources Center of Peking Union Medical College (Beijing, China). OVCAR8 and A2780 cells were propagated in RPMI1640 (HyClone), whereas SKOV3, ID8, and CAOV3 cells were grown in DMEM/F12 or DMEM (HyClone). All culture media contained 10% FBS (Gibco), 50 IU/mL penicillin, and 50 μg/mL streptomycin (Gibco). Cells were incubated at 37°C with 5% CO2 and routinely screened for mycoplasma.

**Assessment of cell proliferation**

To assess proliferation, cells were plated at 5 × 10³ per well in 96-well plates (quintuplicate). Real-time confluence was monitored using the IncuCyte S3 system (Sartorius, Göttingen, Germany), capturing four phase-contrast images per well every 3 h with a 10X objective. Viability was concurrently evaluated via the CellTiter-Glo 3D Assay (Promega), with ATP luminescence normalized to cell counts from imaging. For endpoint viability, a CCK-8 assay (Dojindo, Rockville, MA, USA) was conducted after 48 or 72 h of drug exposure; 10 μL CCK-8 reagent was added per well, and absorbance (450 nm) was recorded 1 h post-incubation using a Tecan Spark multimode reader. GraphPad Prism determined IC50 values, while CompuSyn calculated combination indices (CI < 1 denoting synergy). Representative micrographs were acquired on a Leica system.

**Immunofluorescence analysis**

For confocal microscopy, cells subjected to indicated treatments were fixed in 4% paraformaldehyde, permeabilized, and then blocked using 0.8% BSA solution for 1 h. Subsequently, cells were exposed to primary antibodies (all at 1:50) overnight at 4°C, followed by a 2-h incubation with secondary antibodies (ZSGB-BIO, China). Nuclear counterstaining was carried out using DAPI. Immunofluorescent images were obtained using confocal microscope (FV1000-IX81). Primary antibodies were employed as follows: anti-dsDNA (Proteintech, 85781-2-RR), anti-RAD51 (Abcam, ab176458)

**Western blotting**

Cell lysates were prepared according to standard methods. Proteins were resolved via 10% SDS-PAGE and blotted onto PVDF filters. Following blockade, membranes were probed with target-specific primary antibodies at 4°C overnight, fhen incubated with HRP-linked secondary antibodies (ZB-2301, ZB-2305) for 1 h at ambient temperature. Immunoreactive bands were visualized employing an ECL detection system (P1050-100, Applygen). All antibodies used were commercially validated. We purchased anti-BRCA1 (ab213929), anti-GAPDH (ab181603), and anti-SLC40A1 (ab235166) from Abcam; anti-FLAG (F9291), anti-HA (H6901), and anti-Vinculin (MAB3575) from Sigma-Aldrich; anti-STING (13647), anti-cGAS (79978), anti-Phospho-IκB-α Ser32 (2859), anti-Phospho-TBK1 Ser172 (5483), anti-Phospho-IRF3 S396 (29047), anti-Phospho-IκB-α (2859T), anti-STAT3 (9193), anti-Phospho-STAT3 Tyr705 (9145), and anti-FTH1 (3998) from Cell Signaling Technology; anti-IκB-α (10268-1-AP), anti-TBK1 (28397-1-AP), and anti-IRF3 (11312-1-AP) from Proteintech; anti-GPX4 (A11243) from Abclonal; and anti-PARP1 (436400) from Thermo Fisher.

**Multiplex IHC staining protocol**

OC tissue samples was obtained from Department of Obstetrics and Gynecology, Peking University Third Hospital (Beijing, China). Tissue sections (OVS) underwent dewaxing in xylene followed by graded ethanol rehydration. Multiplex immunohistochemical staining was performed utilizing an Opal 7-color kit (Akoya Bioscience, USA). The first round, the antigen was retrieved by pressure cooking (EDTA pH 9.0) at 125°C for 3 min. Slides were cooled to RT, washed with TBST/0.5% Tween (3 times, 3 min), and incubated with hydrogen peroxide (H2O2, 3%) for 15 min. Slides were washed and blocked with a blocking buffer (VECTASHIELD, USA) for 10 min. Primary antibody, FPN (Novus, NBP1-21502 1:5, dye520), was incubated at RT for 40 min. Slides were washed, and an HRP-conjugated secondary antibody was incubated at RT for 10 min. TSA dye (1:100) was applied for 10 min after washes. For the second and subsequent rounds, antigen retrieval was performed in EDTA buffer (pH 6.0) using a microwave (100-150 mW, 15 min). Nuclei were stained with DAPI (PerkinElmer, USA) and mounted with medium (Solarbio, Beijing, China). Anti-rabbit or anti-mouse secondary antibodies (ZSGB-Bio) were used at a 1:1,000 dilution.

**RNA-seq data processing and analysis**

Cellular RNA was isolated and its quality verified prior to library preparation. mRNA enrichment was achieved through poly(A) selection, after which libraries were constructed via fragmentation, cDNA synthesis, and adapter attachment. Paired-end 150 bp (PE150) sequencing was carried out on an Illumina platform. Adapter sequences were trimmed and low-quality reads (>10% N content or >50% bases below Q20) were discarded to generate clean reads for downstream processing. Clean reads were mapped to the human reference genome (Homo_sapiens. GRCh38_release95) with HISAT2. Transcript-level quantification was computed as FPKM employing StringTie. For differential expression profiling, the DESeq2 R package was utilized, with significance thresholds set at FDR-adjusted p < 0.05 and |log2 fold change| ≥ 1. Enrichment of biological pathways was assessed by Gene Set Enrichment Analysis (GSEA) based on genome-wide log2 fold change values.

**RNA interference (RNAi)**

shRNA plasmids (shNC, shFPN, and shRNA-GFP) were acquired from Ruibo Chem (Shanghai, China). Lentiviral packaging was achieved by co-transfecting HEK293T cells with expression vectors (shFPN/shNC) and packaging plasmids (PLP1/PLP2).The supernatants containing the virus were harvested after 48 h. The OC cells were then infected with the harvested virus and sorted by flow cytometry.

**Statistical analysis**

Results are expressed as mean ± standard error of the mean (S.E.M.). Pairwise comparisons of continuous variables were analyzed using two-tailed Student's t-tests. When more than two groups were compared, one-way ANOVA with Tukey's multiple comparison post hoc test was applied; factorial experiments utilized two-way ANOVA. A P value < 0.05 was considered statistically significant. All statistical computations were carried out using GraphPad Prism software.
